# Knowledge and Prior Use of HIV Self-Testing in Madrid and Barcelona among Men Who Have Sex with Men More than One Year after Its Legal Authorization in Spain

**DOI:** 10.3390/ijerph19031096

**Published:** 2022-01-19

**Authors:** Juan-Miguel Guerras, María-José Belza, María-José Fuster, Luis de la Fuente, Patricia García de Olalla, David Palma, Jorge-Néstor García-Pérez, Juan Hoyos, on behalf of the Methysos Project Group

**Affiliations:** 1 Centro Nacional de Epidemiología, Instituto de Salud Carlos III, 28029 Madrid, Spain; jguerras@isciii.es (J.-M.G.); lfuente@isciii.es (L.d.l.F.); 2 CIBER Epidemiología y Salud Pública (CIBERESP), 28029 Madrid, Spain; polalla@aspb.cat (P.G.d.O.); ext_dpalma@aspb.CAT (D.P.); 3 Escuela Nacional de Sanidad, Instituto de Salud Carlos III, 28029 Madrid, Spain; 4 Sociedad Española Interdisciplinaria del SIDA, SEISIDA, 28036 Madrid, Spain; mjfuster@psi.uned.es; 5 Departamento de Psicología Social y de las Organizaciones, Universidad Nacional de Educación a Distancia, 28040 Madrid, Spain; 6 Servicio de Epidemiología, Agència de Salut Pública de Barcelona, 08023 Barcelona, Spain; 7 Unidad de ITS de Vall d’Hebron-Drassanes, Hospital Vall d’Hebron, 08001 Barcelona, Spain; g.perez@vhebron.net; 8 Independent Researcher, 28013 Madrid, Spain; hoyosmiller@hotmail.com

**Keywords:** early diagnosis, HIV self-testing (HIVST), men who have sex with men (MSM)

## Abstract

This study assessed the knowledge and prior use of HIV self-testing in a sample of men who have sex with men (MSM) recruited in a sexual health clinic and two community-based testing sites in Madrid and Barcelona, >12 months after its legal authorization. Between March 2019 and December 2020, we recruited 2044 MSM. Participants completed a self-administered questionnaire while waiting to be tested for HIV and other STIs. We built two Poisson regression models to assess factors associated with prior knowledge and with use. Among those who had used self-testing in the past we assessed frequency of use and several aspects related to the last testing episode. The proportion of participants that knew about the existence of self-testing and had already used it was of 26.3% and 5.1% respectively. Both, knowledge and use were independently associated with being born in Spain or other western European countries, university education and more recent HIV testing. Additionally, knowledge was associated with older age, having a more favorable economic situation, and not living sexuality in total secrecy. Use was also associated with having received money in exchange for sex. Most (69.5%) reported having self-tested once, 64.8% had self-tested <12 months ago, 63.8% self-tested alone and 71.4% acquired the kit at a pharmacy over the counter. In spite of its authorization and becoming legally available, knowledge and use of HIV self-testing remain low among MSM attending sites specialized in the diagnosis of HIV and other STIs. When designing scale-up strategies, lower levels of knowledge and use in less favored subgroups of MSM need to be factored in.

## 1. Introduction

HIV remains an important public health concern in Western Europe. In Spain, as in the rest of Western Europe, men who have sex with men (MSM) are the group most affected by the epidemic. In 2019, 2.698 new cases of HIV were reported to the Spanish HIV surveillance system, of which 56.6% were acquired through sex between men [1]. One of the main strategies to fight the epidemic is to identify new infections and initiate treatment as early as possible. Early diagnosis and treatment allow individuals to fully benefit from highly active antiretroviral therapy (HAART) by reducing morbidity [2,3] and mortality [4]. Additionally, early detection and HAART initiation are also important as a way of controlling the expansion of the epidemic since HAART greatly reduces the probability of transmission by reducing viral load [5,6]. Promoting early diagnosis and treatment are in fact two of the keystones of the 95-95-95 UNAIDS fast track strategy to end the epidemic. According to it, by 2030, 95% of all individuals living with HIV should be diagnosed, 95% of which should be on treatment and 95% of these should achieve viral suppression [7].

In Spain, HIV testing is offered free of cost at all levels of the national health system and it can also be found anonymously in a network of Sexual Health Clinics [8]. HIV rapid testing is also offered outside clinical settings and can be found in community-based organizations (CBO) [9] and community pharmacies in some regions [10]. However, testing frequency of MSM living in Spain [11] is far from meeting the recommendation of testing at least each 12 months [12]. In fact, 41.3% of the new cases of HIV reported among MSM in 2019 were diagnosed at a late stage of infection (CD4 count of <350 mm^3^) [1]. HIV self-testing, is the most recent testing methodology that has been added to already existing options. In Europe, the United Kingdom authorized HIV self-testing for public use in 2014 and since then it has spread to other European countries [13,14]. In Spain, it has been legally commercialized since December 2017 [14,15]. HIV self-testing requires the user to take a blood or a saliva-based sample, perform the test and interpret the results. When reactive, self-testing requires confirmation testing. It is considered an accurate testing methodology and is supported by the World Health Organization [16].

HIV self-testing could help remove some of the barriers to testing and increase testing frequency in MSM that do not meet testing recommendations. Compared with traditional on-site testing, self-testing offers increased privacy and anonymity and is highly convenient since it does not require appointments or queuing [17]. According to the opinions of MSM recruited in online gay dating websites and apps in Spain, acceptability and potential use of HIV self-testing is high [18] and it is a highly valued option [19]. In fact, several clinical trials also support self-testing’s capacity to increase testing frequency among MSM and other highly vulnerable populations [20,21] but there is little real-life data on its capacity to promote testing. This is challenging since HIV statistics do not reflect the setting where HIV testing is occurring. One way of overcoming this limitation is through self-reported data. In this sense, outcomes related to knowledge and prior use of self-testing are important ways of overcoming this surveillance limitation and to gauge the capacity of this still relatively new testing option to promote testing and increase testing frequency among MSM.

Knowledge and prior use have been previously assessed in Spain. A study conducted between 2010–2012 among attendees of a rapid testing program run by a community-based organization [22], showed low knowledge (5.3%) and near-to-zero (0.6%) use of HIV self-testing. Similarly, an online-based study conducted during 2012 and 2013, reported that under 5% of the participant MSM knew about the existence of self-testing and less than 1% had used it [23]. Another online-based study conducted in MSM in 2016, reported higher knowledge levels (14.5%) [24] but still very low use (1.5%). This same study also presented data on knowledge and use for seven other countries: Belgium, Denmark, Germany, Greece, Portugal, Romania and Slovenia. Knowledge ranged from 15.3% to 35.1% and prior use from 0.1% to 4.5%. Finally, knowledge and use were also assessed in a study conducted among MSM in France [25] which presented a level of knowledge of 30% and a level of prior use of <1%. However, all the aforementioned studies were carried out before the authorization of self-testing in the studied countries. We only found one study that assessed prior -use (but not knowledge) after the authorization of self-testing. In this study, 9.9% of participants (mainly MSM) reported having used it in the past [26].

Thus, there is roughly no information on how the authorization and subsequent commercialization of self-testing kits can affect both outcomes. This gap needs to be filled in order to understand the level of permeation that self-testing has had in MSM after its authorization and to guide decision makers when designing HIV self-testing strategies. Furthermore, it will help to identify subpopulations in need of interventions to promote knowledge and subsequent use.

In the present study, we recruited a sample of MSM in specialized HIV/STI diagnosis contexts in the cities of Madrid and Barcelona after the authorization of HIV self-testing. We aim to determine the level of knowledge of this testing method in the MSM population and its capacity of promoting HIV testing by evaluating prior use. Additionally, among those who reported having used it in the past we describe the frequency of past use, time since last self-test, and several characteristics related to their last testing episode.

## 2. Materials and Methods

### 2.1. Participants

A total of 2044 participants seeking to be tested for HIV were recruited between March 2019 and December 2020 in the cities of Madrid and Barcelona which are the two largest cities in Spain with 3,334,730 and 1,664,182 inhabitants respectively [27]. In order to be included in the study, participants needed to report having had anal sex with another man at least once in their life and either having a negative result in their last HIV testing episode or not having tested for HIV in the past. HIV-positive individuals, women, men with no previous sexual experience or who reported having had sex exclusively with women were not included in the study. We also did not include individuals under 18 years old.

### 2.2. Recruitment and Data Collection Instrument

Recruitment was carried out in three sites. In Barcelona, it was conducted in the largest sexual health clinic in the city (Drassanes) and in a community-based program run by the Public Health Agency of Barcelona. In Madrid, it was conducted in a community-based program run by the Pink Peace non-governmental association. Sexual health clinics in Spain offer on-demand services and perform traditional testing for all STIs. Community programs, offer rapid testing for HIV, syphilis, and sometimes Hepatitis C. They also carry out active recruitment through ads and profiles inviting users of dating apps to visit their program for testing.

Attendees were approached for participation while waiting to be tested in the recruitment sites. Those who agreed to participate, answered a self-administered online questionnaire on a tablet. The questionnaire included questions that assessed a number of areas including sociodemography, openness, sexual behaviors, history of STI, and history of HIV testing. It also included a section that assessed several aspects related to HIV self-testing including knowledge about the existence of HIV self-testing and prior use which are the two main outcomes of the present paper. Knowledge was assessed with the question “Did you know that in SPAIN for more than a year now, you can buy an HIV self-test in pharmacies and para-pharmacies without needing a prescription?”. Participants could choose between three closed response options: No, I did not; I had heard something, but I was not sure; Yes, I knew. Use was assessed with the question “Have you ever been tested for HIV using a self-testing kit”. Participants had to answer “yes” or “no”. Those who answered having used it in the past, were inquired about the number of times they had used it, time since last use, place of acquisition of last self-testing kit and with whom did he perform the last self-test. The study was approved by the Research Ethics Committee of the Instituto de Salud Carlos III (CEI PI 44_2018_subproyecto1-v2 and CEI PI 44_2018_subproyecto2).

### 2.3. Data Analysis

Our study sample, was comprised of 2044 MSM who answered the questions on knowledge and past use of HIV self-testing. We first performed a descriptive analysis of the main characteristics of the sample. Secondly, we estimated the proportion of participants who reported knowledge and past use of HIV self-testing by relevant independent variables. The outcome on the knowledge about the existence of HIV self-testing was collapsed in a two-category variable: (1) Yes; (2) No, I did not; I had heard something, but I was not sure. To estimate the factors associated with knowledge and with past use of self-testing, we used Poisson regression with robust variance in the framework of generalized linear models’ analysis [28,29] to calculate the crude and adjusted prevalence ratios (PRs) with their 95% confidence intervals (CI95%). We initially included all the relevant variables with a significance level ≤ 0.20 and employed the minimum Akaike information criterion and the minimum Bayesian Schwartz information criterion for model comparisons and to select the optimal model. Final models were adjusted by age. Finally, for those who reported having used an HIV self-test in the past, we assessed the number of times they had used it, time since last use, place of acquisition and person with who they used it the last time.

## 3. Results

### 3.1. Participants’ Main Characteristics

Approximately half (46.4%) of all participants were recruited in the Madrid community-based program, 36.2% were under 30 years of age, 58.0% were born in Spain, 59.1% had finished their university education, 58.8% had a comfortable or good economic situation, and 78.6% reported living in a city of more than 1,000,000 inhabitants. Approximately six in ten (61.2%) reported living their sex life with other men in a completely open manner and 63.2% reported only having had sex with other men. One in four MSM (25.6%) reported not having had unprotected receptive anal sex with another man during the last 12 months, 8.9% reported having paid and 12.4% reported having been paid for sex. Only 5.8% reported having had unprotected intercourse with women in the last 12 months. Regarding history of STI, 56.2% reported a previous diagnosis (29.3% during the previous 12 months). Only 6.0% reported not having been tested for HIV before and 77.1% had been tested in the previous 12 months (47.6% in the previous 6 months) (Table 1).

### 3.2. Knowledge and Past Use of HIV Self-Testing

Overall, 26.3% reported knowing about the existence of this testing option and 5.1% reported having used it in the past.

The proportion of MSM who reported knowing about the existence of HIV self-testing as well as the crude and adjusted PRs are presented in Table 2. Knowledge was higher among participants older than 30 (30–39 years Adj PR = 1.2 (CI95%: 1.0–1.4); ≥40 Adj PR 1.2 (CI95%: 1.0–1.5)); those who were born in Spain (Adj PR = 1.8 (CI95%: 1.5–2.2)) or in other western European countries (Adj PR = 1.5 (CI95%: 1.1–2.1)); those who had completed university education (Adj PR = 1.2 (CI95%: 1.0–1.4)) and among those who had a comfortable economic situation (Adj PR = 1.4 (CI95%: 1.0–1.9)). Compared with men who lived their sex life hidden or in total secrecy, knowledge was higher among those who reported living it discreetly (Adj PR = 1.5 (CI95%: 1.0–2.5)) or openly (PR = 1.9 (CI95%: 1.2–3.0)). Compared with MSM with no previous HIV testing experience, knowledge was higher among those whose last test was >5 years ago (Adj PR = 2.3 (CI95%: 1.2–4.7)); 6–12 months ago (Adj PR = 1.8 (CI95%: 1.1–3.2)) and less than 6 months ago (Adj PR = 2.4 (CI95%: 1.2–4.7)).

Prevalence of use by relevant independent variables is presented in Table 3. Compared with Latin-American MSM, use was higher among Spaniards (Adj PR = 1.6 (CI95%: 1.0–2.5)) and those born in other western European countries (Adj PR = 2.4 (CI95%: 1.3–4.6)). Use was also higher in those who had completed their university education (Adj PR = 1.5 (CI95%: 1.0–2.3)), those who reported having been paid for sex (Adj PR = 1.8 (CI95%: 1.1–2.9)) and among those who received their last test less than six months ago (Adj PR = 2.5 (CI95%: 1.4–4.6)).

Among those who reported having previously used a self-test, 69.5% reported having used it only once, 64.8% used one in the last 12 months and 71.4% bought the last one over the counter at a pharmacy. Almost two in three (63.8%) conducted their last self-test alone (Table 4).

## 4. Discussion

In this sample comprised of MSM recruited at a sexual health clinic and two community-based counseling and testing programs after the authorization of HIV self-testing, knowledge and use remain low. Higher knowledge was found in older MSM, those born in Spain or in other western European countries, with university education, with a comfortable economic situation, and whose sexual life with other men was not completely hidden. It was also higher among those who reported having been tested before. Prior use was also associated with having been born in Spain or western Europe, having university education and having previous HIV testing experience. Additionally, it was also associated with having been paid for sex in the last 12 months. Self-tests were generally acquired over the counter at pharmacies, use was recent, infrequent and tended to be used alone.

Compared with the studies conducted previously in Spain, the level of knowledge in our sample was nearly two times higher than in the most recent study [24] and approximately five times higher than in the two older studies [22,23]. When compared with other countries, we found that our level of knowledge was slightly lower than in other countries such as France [25], Germany or Belgium [24]. Regarding use, the level of previous use we found in our sample was >6 times higher than in the older studies conducted in Spain [22,23] and nearly 3.5 times higher than in the most recent study [24]. When compared with other countries use was >6 times higher than in France [25] and at least two times higher than in the rest of the countries assessed in the multi-country study [24] with the exception of Germany where level of use was similar to the one found in our sample. Use was half of that reported by the only study that was conducted after self-testings’ legal authorization in France [26].

Differences of knowledge with the studies conducted in Spain could be explained by different population characteristics derived from different recruitment methods (online vs. on site) [23,24]. Our sample was comprised of MSM recruited in Madrid and Barcelona both of which are LGBTQIA+ friendly cities. Whether levels of knowledge in smaller cities and rural areas could be different remains unknown but needs to be assessed. These MSM subpopulations—although smaller in size—could face unique barriers to access testing and self-testing could contribute to alleviating them. Having said this, participants who reported living in municipalities of under 1,000,000 inhabitants presented similar knowledge rates and this variable did not reach the necessary < 0.20 significance in the crude analysis to be considered in the adjusted multivariable model. On the other hand, differences could also be because the recruitment for this study was conducted after the authorization of Self-testing as opposed to the rest of the studies conducted in Spain [22,23,24].

The higher levels of knowledge found in other European countries [24,25]—where studies were also conducted before the authorization of self-testing—suggest that baseline knowledge at the moment of authorization was lower in Spain than in countries such as Belgium or France. Knowledge is important because it is a necessary condition for use. In Spain, authorization was followed by coverage both in general and gay-oriented media which probably explains the increase in knowledge when compared with previous studies. However, the proportion of participants who knew about it remained low. No specific campaign was conducted at the moment of its authorization by the ministry of health and could be considered at this point if levels of knowledge want to be raised. Other stakeholders such as CBO/NGOs, clinicians and public health professionals could also play a role in the promotion of self-testing but their position towards this testing option—especially among CBO/NGO workers—is less favorable than among MSM mainly because they consider the presence of an expert to counsel and inform about the result to be essential [30].

Knowledge was higher among participants of older age which is a promising result since surveillance data in Spain reports that delayed diagnosis increases with age [1]. High rates of late diagnosis among older individuals suggest that they are in need of alternative testing methods to allow them more frequent testing and earlier diagnosis. However, this association did not hold in the multivariable model for self-testing use, suggesting the need to find adequate strategies to transform higher knowledge into higher use in older age groups. Knowledge was also higher among socially favored MSM (i.e., individuals from Spain and other western European countries, with university education and with a comfortable economic situation) which is different from what was found in previous findings in Spain where increased knowledge rates were not found in more favored subgroups [22,23]. This could indicate that self-testing is finding its way into society through higher socioeconomic stratums. Likewise, knowledge was lower among those who lived their sexuality in total secrecy. Not being open about one’s sexuality is one of the factors commonly associated with not having been tested for HIV [31,32,33]. The privacy and anonymity offered by self-testing could facilitate testing to this subpopulation as suggested in other studies [34] and strategies should be put in place to inform them about the existence of a testing approach that allows checking HIV serostatus without disclosing sexual behavior. Finally, knowledge tended to be higher among those who had previous testing history (except for those who reported having received their last test between 1 and 5 years ago). Having been tested in the past has also been found to be associated with knowledge in previous studies [22,23].

Regarding use, only 5% of our participants reported having used it in the past. This is still a low proportion that needs to be raised in order to make a real contribution in the first “95” of the UNAIDS strategy. However, we need to keep in mind that recruitment started only 15 months after the authorization of HIV self-testing and, as aforementioned, knowledge was still low. In fact, the number of participants that reported more than one self-test is low and last reported use was recent. Similar to knowledge, levels of use of HIV self-testing could be different in MSM living in smaller cities and rural areas and needs to be assessed in samples with a larger presence of these subpopulations. However, in the present study, participants living in municipalities of under 1,000,000 inhabitants, presented similar levels of use. Even if no additional information campaigns are carried out, knowledge will tend to increase through word of mouth and will naturally lead to higher overall use. Further studies should be carried out to identify groups where knowledge is translating into use. This would imply restricting multivariable analyses assessing use to those who already know about the existence of HIV self-testing.

When considering how to increase the use of self-testing, we need to take into account distribution modalities. In our sample, self-testing kits were acquired mainly over the counter in a pharmacy. Promoting web-based distribution strategies could increase convenience even further and according to a recent meta-analysis, increases the probabilities of self-testing uptake [35].

It is important to note that, similar to knowledge, use was lower in some less favored subgroups (i.e., Latin-American migrants, with no university education). When promoting HIV self-testing within these subpopulations, the cost needs to be especially factored in [36]. Currently, the price of self-testing kits is 25–30 euros. This price could be too high for MSM of lower socioeconomic status. With time, prices could fall due to competition of new self-testing kits introduced in the market by pharmaceutical companies but as of now, if benefits derived from HIV self-testing need to reach less favored subgroups, roll-out strategies need to consider the cost. Programs offering subsidies, discounts, or free distribution of self-testing kits directly targeting immigrants and less socially favored MSM could increase the use of this testing modality and need to be evaluated. Higher use was also reported among those who reported having been paid for sex in the last 12 months which suggests that HIV self-testing is an attractive option for this especially vulnerable population. For male sex workers, HIV self-testing offers the opportunity of checking partners’ HIV serostatus before sexual intercourse with a new partner and can be incorporated as an additional tool to bio-behavioral prevention. However, in our sample this type of use was rare with very few participants reporting having used HIV self-testing with their occasional sex partners and most of our participants reported having used it alone. The reasons for not using self-testing kits were not explored although previous works report that aspects such as concerns about reliability or fear of not being able to perform a self-test properly are not within the main preoccupations of MSM [24].

Results are not without limitations. When assessing unprotected anal intercourse, we only asked about receptive anal sex which is the role that implies the highest risk for HIV. We do not know if also including insertive anal sex in the assessment of the association between unprotected anal intercourse and the main outcomes could have implied the association of this variable with knowledge and use in the final multivariable models. This is a convenience sample and therefore generalizations to the overall MSM population need to be made with caution. Our sample is consists of individuals residing in large cities of over 1,000,000 inhabitants and is lacking participants living in smaller cities and rural areas that probably face particular barriers to access HIV testing services. There is also a relevant selection bias that needs to be noted: by definition, all participant MSM were searching for an HIV test and therefore knew how to access existing testing services (in this case community-based programs or sexual health clinics). For this study population, self-testing could improve convenience and result in an increase in testing frequency among high-risk individuals. However, it does not include MSM with no previous testing experience or those who are not actively seeking to be tested. It is essential that HIV-testing strategies reach such populations and self-testing could be a way of targeting them. Further studies with different recruitment methods need to be conducted to assess knowledge and use for MSM with no previous testing experience who are not actively seeking an HIV test, and among those living in smaller cities and rural areas. Finally, other studies will also need to be conducted to assess knowledge and use of HIV self-testing in other highly vulnerable MSM not assessed in the present paper such as those involved in sexualized drug use or chemsex [37].

## 5. Conclusions

Knowledge and use of HIV self-testing after the authorization of this testing option by Spanish authorities remains low in a sample of MSM recruited in contexts specialized in the diagnosis of HIV and other STIs. Knowledge about self-testing needs to be promoted drastically as a necessary first step to scale up self-testing. Additionally, the cost and distribution methods of self-testing kits need to be taken into account in order to reach socially and economically less favored MSM as well as those who do not live their sexuality in an open manner who, in this study, reported lower knowledge and use.

## Figures and Tables

**Table 1:** Main characteristics of men who have sex with men recruited in Barcelona and Madrid (*n* = 2044).

	*n*	%
**Recruitment site**		
Madrid community-based program	949	46.4
Barcelona community-based program	678	33.2
Barcelona sexual health clinic	417	20.4
**Age**		
<30	740	36.2
30–39	722	35.3
≥40	582	28.5
**Place of birth**		
Latin America	613	30.0
Spain	1185	58.0
Western Europe	146	7.1
Others	100	4.9
**What is the highest level of education you have completed?**		
Up to primary school	144	7.1
Up to upper secondary	687	33.8
University education	1203	59.1
**Self-defined economic situation**		
Difficult/very difficult	201	9.8
Tight	640	31.4
Comfortable/good	1200	58.8
**>1,000,000 inhabitants of place of residency**	1601	78.6
**Lives sex life with other men…**		
Hidden/in total secrecy	122	6.0
Discreetly	669	32.8
Openly	1247	61.2
**Has had sex only with men (ever)**	1291	63.2
Number of men with whom he had unprotected receptive anal sex *		
0	447	25.6
1	582	33.4
2–4	503	28.9
≥5	211	12.1
Unprotected intercourse with women *	119	5.8
Has paid for sex *	181	8.9
Has been paid for sex *	253	12.4
**History of sexually transmitted infections diagnosis**		
No STI diagnosis	887	43.8
STI diagnosis > 12 months ago	543	26.8
STI diagnosis in the last 12 months	594	29.3
**Time since last HIV test**		
Never tested before	123	6.0
More than 5 years	49	2.4
1–5 years	296	14.5
6–12 months	602	29.5
<6 months	972	47.6

* In the last 12 months.

**Table 2:** Proportion of participants who know about the existence of HIV self-testing and associated variables (crude and adjusted prevalence ratios).

Total	Yes		No/Not Sure		CPR *	*p*-Value	CI95% **	APR ***	CI95%
	*n*	%	*n*	%					
	537	26.3	1507	73.7					
**Recruitment site**						^2^			
Madrid community-based program	226	23.8	723	76.2	ref				
Barcelona community-based program	175	25.8	503	74.2	1.1		0.9–1.3		
Barcelona sexual health clinic	136	32.6	281	67.4	1.4		1.1–1.7		
**Age**						^2^			
<30	164	22.2	576	77.8	ref			ref	
30–39	201	27.8	521	72.2	1.3		1.0–1.5	1.2	1.0–1.4
≥40	172	29.6	410	70.4	1.3		1.1–1.7	1.2	1.0–1.5
**Place of birth**						^1^			
Latin America	99	16.2	514	83.8	ref			ref	
Spain	375	31.6	810	68.4	2.0		1.6–2.4	1.8	1.5–2.2
Western Europe	44	30.1	102	69.9	1.9		1.3–2.7	1.5	1.1–2.1
Others	19	19.0	81	81.0	1.2		0.7–1.9	1.1	0.7–7.1
**What is the highest level of education you have completed?**						^1^			
Below university education	179	21.5	652	78.5	ref			ref	
University education	355	29.5	848	70.5	1.4		1.1–1.6	1.2	1.0–1.4
**Self-defined economic situation**						^1^			
Difficult/very difficult	33	16.4	168	83.6	ref			ref	
Tight	138	21.6	502	78.4	1.3		0.9–1.9	1.1	0.8–1.5
Comfortable/good	365	30.4	835	69.6	1.9		1.3–2.6	1.4	1.0–1.9
**Lives sex life with other men…**						^1^			
Hidden/in total secrecy	16	13.1	106	86.9	ref			ref	
Discreetly	149	22.3	520	77.7	1.7		1.0–2.8	1.5	1.0–2.5
Openly	371	29.8	876	70.2	2.3		1.4–3.7	1.9	1.2–3.0
**Sex of lifetime sexual partners**						^3^			
Men and women	180	23.9	573	76.1	ref				
Only men	357	27.7	934	72.3	1.2		1.0–1.4		
**Has been paid for sex (last 12 months)**						^2^			
Yes	51	20.2	202	79.8	ref				
No	486	27.2	1304	72.8	1.3		1.0–1.8		
**Unprotected intercourse with women (last 12 months)**						^2^			
Yes	19	16.0	100	84.0	ref				
No	518	26.9	1406	73.1	1.7		1.1–2.7		
**Time since last HIV test**						^1^			
Never tested before	13	10.6	110	89.4	ref			ref	
More than 5 years	14	28.6	35	71.4	2.7		1.3–5.8	2.3	1.2–4.7
1–5 years	64	21.6	232	78.4	2.0		1.1–3.7	1.6	0.9–2.8
6–12 months	149	24.8	453	75.2	2.3		1.3–4.1	1.8	1.1–3.2
<6 months	297	30.6	675	69.4	2.9		1.7–5.0	2.4	1.4–4.0

The table shows results for variables with a significance level of ≤0.20 in the crude analysis and variables retained in the final model. * Crude prevalence ratio, ** 95% confidence interval, *** adjusted prevalence ratio. ^1^ *p*-value < 0.01; ^2^ *p*-value < 0.05; ^3^ *p*-value ≤ 0.20.

**Table 3:** Proportion of participants who reported having used a self-testing kit and associated variables (crude and adjusted prevalence ratios).

Total	Yes		No		CPR *	*p*-Value	CI95% **	APR ***	CI95%
	*n*	%	*n*	%					
	105	5.1	1939	94.9					
**Age**						^4^			
<30	33	4.5	707	95.5					
30–39	43	6.0	679	94.0					
≥40	29	5.0	553	95.0					
**Place of birth**						^3^			
Latin America	24	3.9	589	96.1	ref			ref	
Spain	64	5.4	1121	94.6	1.4		0.9–2.2	1.6	1.0–2.5
Western Europe	14	9.6	132	90.4	2.5		1.3–4.7	2.4	1.3–4.6
Others	3	3.0	97	97.0	0.8		0.2–2.5	0.8	0.3–2.7
**What is the highest level of education you have completed?**						^2^			
Below university education	32	3.8	799	96.2	ref			ref	
University education	72	6.0	1131	94.0	1.6		1.0–2.4	1.5	1.0–2.3
**Self-defined economic situation**						^3^			
Difficult/very difficult	6	3.0	195	97.0	ref				
Tight	28	4.4	612	95.6	1.5		0.6–3.5		
Comfortable/good	71	5.9	1129	94.1	2.0		0.9–4.5		
**Has been paid for sex (last 12 months)**						^3^			
No	87	4.9	1703	95.1	ref			ref	
Yes	18	7.1	235	92.9	1.5		0.9–2.4	1.8	1.1–2.9
**Time since last HIV test**						^1^			
>1 years-never tested	12	2.6	456	94.8	ref			ref	
6–12 months	25	4.1	577	95.9	1.6		0.8–3.2	1.5	0.8–3.0
<6 months	68	7.0	904	93.0	2.7		1.5–5.0	2.5	1.4–4.6

The table shows results for variables with a significance level of ≤0.20 in the crude analysis and variables retained in the final model. * Crude prevalence ratio, ** 95% confidence interval, *** adjusted prevalence ratio. ^1^ *p*-value < 0.01; ^2^ *p*-value < 0.05; ^3^ *p*-value ≤ 0.20; ^4^ *p*-value > 0.20.

**Table 4:** Number of self-tests performed, time since last self-test, place of acquisition and companion during last testing episode among participants who had self-tested at least once (*n* = 105).

	*n*	%
**Number self-testing kits used**		
One	73	69.5
Two	12	11.4
Three or more	20	19.0
**Time since last self-test**		
<3 months	15	14.3
3 to 12 months	53	50.5
>12 to 24 months	26	24.8
>24 months ago	11	10.5
**Where was the last self-test acquired**		
At a pharmacy (over the counter)	75	71.4
At an online pharmacy	7	6.7
At a country (other than Spain) where it is legally sold	10	9.5
Purchased it on the internet without having certainty about its legality	1	1.0
Others	12	11.4
**With whom did he perform his last self-test**		
Alone	67	63.8
Stable partner	14	13.3
Occasional partner	4	3.8
Friend	13	12.4
CBO/NGO staff	2	1.9
Health professional	5	4.8

## Data Availability

The datasets used and/or analyzed during the current study are available from the corresponding author on reasonable request.

## Supplementary Materials

The following supporting information can be downloaded at: https://www.mdpi.com/article/10.3390/ijerph19031096/s1, File S1: List of the Collaborators/Membership of the Group.
