# Supplementary material for: Knowledge and Prior Use of HIV Self-Testing in Madrid and Barcelona among Men Who Have Sex with Men More than One Year after Its Legal Authorization in Spain
Source: Int J Environ Res Public Health. 2022 Jan 19;19(3):1096. doi: 10.3390/ijerph19031096 (PMC8834423; doi:10.3390/ijerph19031096)
Supplement: Supplementary file 1 [file ijerph-19-01096-s001.zip › ijerph-1498625-supplementary.pdf]

List of the Collaborators/Membership of Methysos Project Group:

Marta Donat, María del Carmen Burgos, and César Pérez Romero *(Instituto de Salud Carlos III, Madrid);*

José Antonio San Juan Bueno *(Asociación Pink Peace, Madrid);*

Francisca Roman Urrestaruzo, Jesus E Ospina, and Miguel Alarcón Gutiérrez *(Agència de Salut Pública de Barcelona, Barcelona);*

Jorge del Romero; Oskar Ayerdi, Carmen Rodríguez, Sonsoles del Corral Del Campo, Natividad Jerez Zamora, Marta Ruiz Fernández, and Montserrat González Polo *(Centro Sanitario Sandoval, Madrid);*

and María Jesús Barbera Gracia, Luis López Pérez, Claudia Broto Cortes, and Julio Morais Martin *(CAP Drassanes, Barcelona).*
